# *The possible short-term of Nigella sativa – L* in the management of adolescent polycystic ovarian syndrome: results of a randomized controlled trial

**DOI:** 10.1186/s13048-024-01460-x

**Published:** 2024-07-12

**Authors:** Azamsadat Mahmoudian, Akram Ashouri, Roghaieh Rahmani Bilandi, Fatemeh Mohammadzadeh, Sareh Dashti, Narjes Bahri

**Affiliations:** 1 Department of Obstetrics & Gynecology, School of Medicine, Social Determinants of Health Research Center, Gonabad University of Medical Sciences, Gonabad, Iran; 2 Department of Midwifery, School of Medicine, Social Determinants of Health Research Center, Gonabad University of Medical Sciences, Gonabad, Iran; 3 Department of Midwifery, Faculty of Medicine, Reproductive Health and Population Research Center, Gonabad University of Medical Sciences, Gonabad, Iran; 4 Department of Epidemiology and Biostatistics, School of Health, Gonabad University of Medical Sciences, Gonabad, Iran; 5 Department of Midwifery, Faculty of Nursing and Midwifery, Mashhad Medical Sciences, Islamic Azad University, Mashhad, Iran; 6 Department of Public Health, Faculty of Paramedicine, Mashhad Medical Sciences, Islamic Azad University, Mashhad, Iran

**Keywords:** Polycystic ovary syndrome, Nigella sativa, Medroxyprogesterone, Adolescent

## Abstract

**Background:**

Polycystic ovary syndrome (PCOS) is the most common endocrine disorder in reproductive age and the most common cause of infertility due to anovulation. PCOS in adolescents is concerning. Nigella sativa is effective in improving gonadotropins and sex hormones. The current study was designed to investigate the effect of Nigella sativa supplementation on PCOS symptoms and their severity in adolescents.

**Methods:**

The current randomized clinical trial was conducted on 114 adolescents with PCOS who were referred to gynecologist offices and clinics in Gonabad, Iran from March 2022 to March 2023. Participants were randomly allocated to the intervention (Nigella sativa 1000 mg/day) and control (10 mg/day medroxyprogesterone from the 14th day of the cycle for 10 nights) groups. The study duration was 16 weeks. Ovarian volume (measured by ultrasound), anthropometric and blood pressure; serum testosterone, dehydroepiandrosterone (DHEA), dehydroepiandrosterone sulfate (DHEA-S), luteinizing hormone (LH), hirsutism severity (Ferriman–Gallwey score) levels were evaluated before and after the study.

**Results:**

Data from 103 participants (control group = 53, intervention group = 50) were analyzed. The mean age of participants was 17.0 (Interquartile range [IQR]:2.0). The mean difference in hirsutism score changes (*p* < 0.001), right (*p* = 0.002), and left (*p* = 0.010) ovarian volume, serum LH (*p* < 0.001) and testosterone (*p* = 0.001) were significantly higher in the intervention group compared to the control group. The frequency of oligomenorrhea, menometrorrhagia, and amenorrhea, were significantly reduced after the study in the intervention group compared to the control group (*p*s < 0.001).

**Conclusions:**

Short-term Nigella sativa supplementation may be effective in reducing ovarian volume and improving hormonal balance, and menstrual irregularities in adolescents with PCOS. Further research and long-term studies are warranted to validate the potential therapeutic effects of Nigella sativa in adolescents with PCOS.

**IRCT registration number:**

IRCT20221017056209N1 Registration date: 2022-11-22.

**Supplementary Information:**

The online version contains supplementary material available at 10.1186/s13048-024-01460-x.

## Background

Polycystic ovary syndrome (PCOS) is the most common endocrine disorder among women of reproductive age and is the most common cause of infertility due to anovulation [1, 2]. In 2022, the global prevalence of PCOS based on Rotterdam criteria ranged between 2.2 and 22.5% [3]. In Iran, the prevalence of PCOS based on Rotterdam criteria and sonographic methods were estimated to be 19.5 and 41.1% [6]. PCOS is a complex multifactorial, polygenic, and hereditary disorder and is characterized by hyperandrogenism, polycystic ovaries, and ovarian dysfunction [4]. Short and long-term complications of PCOS impose a great health and economic burden on countries [5].

In recent years, teenagers have been recognized as a susceptible age group to PCOS [1]. Different prevalence rates have been reported for PCOS among teenagers. The reason for this difference may be related to the use of different diagnostic criteria [1]. In a meta-analysis study published in 2019, the global prevalence of PCOs based on Rotterdam criteria among adolescents was 11.4% [1]. Early diagnosis and treatment of adolescents with PCOS can prevent long-term reproductive, cardio-metabolic, and emotional consequences associated with this syndrome [6, 7]. PCOS can lead to the cessation of the menstrual cycle or primary or secondary amenorrhea and secondary infertility [8, 9]. PCOS is also associated with the risk of depression, anxiety, obsessive-compulsive disorder, and physical abuse in adolescents [10].

Several treatment methods have been suggested for adult women with PCOS. Combined hormonal contraceptive (CHC) is suggested for the treatment of menstrual disorders and hyperandrogenism in PCOS [11]. Weight loss is the first non-pharmacological treatment for infertility in obese women with PCOS, while clomiphene citrate (anti-estrogen), aromatase inhibitors (estrone and estradiol reducing), gonadotropins (stimulating ovulation) and metformin (lowering blood sugar) can be effective pharmacological treatments for infertility due to PCOS [12]. However, there is limited information on the efficacy of these treatments in improving the manifestations of PCOS in adolescence [12].

The side effects of pharmacological agents have resulted in the desire to use herbal medicines, especially among women [13]. Medicinal plants are cheap, accessible, and have fewer side effects compared to chemical drugs [14]. Many herbal medicines, including vitex, Cichorium intybus, beetroot, green tea, Foeniculum vulgare Mill, Carum carvi, aloe vera, Stachys lavandulifolia, Nigella sativa, and licorice, have been studied in the treatment of PCOS [15].

Nigella sativa Linn, commonly known as black seed, is a member of the Ranunculaceae family. Nigella sativa has been used for therapeutic purposes in India, Arab countries, Europe and Iran. Nigella sativa compounds include estrone, saponin, phenolic compounds, alkaloids, fatty acids, and volatile oils. Eight types of amino acids are known in the proteins found in Nigella sativa. Nigella sativa contains monosaccharides in the form of glucose, rhamnose, xylose, and arabinose [16]. Nigella sativa has relaxant, fat-lowering, antioxidant, anti-inflammatory, antidiabetic, antihypertensive, antibacterial, analgesic, antiparasitic, anti-flatulent, diuretic, lactating, and antiparasitic, and liver, kidney, blood vessels protective properties [17–19]. It has also been used as a spice and in the treatment of asthma, hypertension, diabetes, inflammation, cough, bronchitis, headache, eczema, fever, dizziness and flu [19]. Some studies have evaluated the effects of Nigella sativa in the treatment of PCOS in adults [16, 17, 19, 20]. Based on the findings of these studies, Nigella sativa can have therapeutic effects on PCOS due to its antioxidant, and insulin resistance-lowering properties [16, 17, 19, 20].

To the best of our knowledge, the effects of Nigella sativa have not been studied among adolescents with PCOS. Considering the known effects of Nigella sativa on adults with PCOS and PCOS-related infertility, the current study was designed to compare the effects of Nigella sativa with medroxyprogesterone acetate on the symptoms of PCOS and their severity in adolescent girls.

## Methods

### Study design, participants, and sampling

The current study was a randomized controlled clinical trial, registered in the Iranian Registry of Clinical Trials (Code: IRCT20221017056209N1. Registration date: 2022-11-22). The study design was based on the Consolidated Standards of Reporting Trials (CONSORT) checklist [21].

The sample size for the study was calculated using the G*Power 3.1.9.2 software considering the type 1 error of 0.01 and the power of 0.99 and the difference in ovarian volume reported among women with PCOS (15.15 ± 5.6 cm^3^) reported in a previous study [22]. The calculated sample size (49 participants in each group) was increased to 57 participants in each group considering 15% dropout.

Participants were selected using a convenience sampling technique from adolescent girls referred to private or governmental Obstetrics and Gynecology clinics in Gonabad, Iran, from March 2022 to March 2023. The inclusion criteria were age range of 12 to 18 years, menstruation history for at least two years, willingness to participate in the study, documented diagnosis of PCOS based on the recent guidelines for the diagnosis of PCOS among adolescents [23] (diagnosis of PCOS was confirmed by a gynecologist), absence of other causes of hyperandrogenism, including non-classic congenital adrenal hyperplasia, body mass index (BMI) between 19 and 25 kg/m^2^, no history of consuming hormonal medications in the past three months, no history of consuming medications that interfere with medroxyprogesterone, including amphetamines, thyroid medications, diphenhydramine, ciprofloxacin, duloxetine, estradiol, omega-3 fatty acids, ecitalopram, pregabalin, levonorgestrel, montelukast, testosterone, cyanocobalamin, ascorbic acid, multivitamins, sertraline, and cetirizine, no allergy to Nigella sativa or medroxyprogesterone based on self-report, no history for hypothyroidism, hyperprolactinemia, or renal, cardiac, hepatic, or bone disorders, hypophysis tumors, cancer or diabetes based on self-report and history taking, no history for smoking or drug abuse, no history of surgery on one or both ovaries, and no history for incidents that trigger psychologic tensions, including death of a relative, accidents, sexual insult, or rubbery, in the past three months. The exclusion criteria were refusal to continue the study, allergic reactions to the intervention medication based on self-report or family member report, lack of adherence to the prescribed medication or supplement (not using the capsules or pills for more than four weeks consecutively or 30% of the capsules or pills during the study).

### Randomization and blinding

Participants were randomly assigned to the intervention group (Nigella sativa) or the control group (medroxyprogesterone) using permutation blocks of sizes 2 and 4. The random allocation sequence was generated using the online website (https://www.sealedenvelope.com) and concealed in sequentially numbered, opaque, sealed envelopes. These envelopes were opened sequentially only after the participant provided informed consent and entered the study.

Only the statistical analyzer was blinded. Treatments were coded as A and B in the dataset to maintain confidentiality and prevent bias during analysis.

### Study instruments

- **Demographic and menstrual questionnaire**: A researcher-made questionnaire was used to obtain the demographic characteristics of the participants. These characteristics included age, education level, maternal education, maternal occupation, paternal education, paternal occupation, child order in the family, and number of siblings. Menstrual history information was obtained based on interviews with the participants and their parents. Menstrual history data included age at menarche, history of dysmenorrhea, duration of menstrual cycle, duration of menstrual bleeding, volume of menstrual bleeding, presence of menstrual disorders, and duration of PCOS diagnosis.
- **Ultrasound assessment**: Ovarian volume was assessed trans-abdominally using an ultrasound device by a radiologist. Ovarian volume was measured in the longitudinal plane by measuring the distance between the inner and outer margins of the ovaries. The ovarian volume was separately recorded for each side. All ultrasound assessments were done by one radiologist.
- **Laboratory measurements**: Serum dehydroepiandrosterone (DHEA), dehydroepiandrosterone sulfate (DHEA-S), serum testosterone, and luteinizing hormone (LH) were measured using the quantitative luminescence method (LIAISON Assay, Diasorin, Dietzenbach, Germany).
- **Anthropometric measurements**: The height, waist, and hip circumference of the participants were measured using a measuring tape to the nearest 0.1 centimeters. The weight of the participants was measured using a digital weighing scale to the nearest 100 g. BMI was then calculated by dividing the weight in kg by the square of height in meters. Waist-hip ratio (WHR) was calculated by dividing the waist circumference to the waist circumference.
- **The Ferriman–Gallwey score**: The Ferriman-Gallwey score was utilized to assess hirsutism among the participants in the study. This scoring system is based on the evaluation of hair density in nine different body areas, such as the upper lip, chin, chest, upper and lower abdomen, thighs, and upper and lower back. The score can range from 0 (indicating no hair growth) to 4 (indicating extensive hair growth) in each area. A total score of 8 or above was indicative of hirsutism [24].

### Study procedure

After obtaining informed consent, the study questionnaires were filled in an interview and the anthropometric measurements were performed in a private, calm, and stress-free environment. The ovarian volume was measured by ultrasound scan, and venous blood samples were taken for laboratory measurements after filling out the questionnaires. Then the participants were allocated to the intervention (Nigella sativa) and control (medroxyprogesterone).

The participants in the intervention group received 1000 mg capsules containing Nigella sativa extract (Barij Essence Pharmaceutical Company, Kashan, Iran) daily for 16 weeks (Supplementary file 1) (55). The product was commercially available in the pharmaceutical market of Iran and has approval from the Iran Food and Drug Organization. The participants in the control group received 10 mg medroxyprogesterone tablets (Aburaihan Pharmaceutical company, Iran) 10 nights per month from the 14th day of their menstrual cycle for 16 weeks [3].

Participants were asked to fill out a logbook for medication consumption and report any side effects to the primary researcher. Participants in both groups were reminded of regular consumption of the drugs through short messaging system (SMS) reminders daily.

At the end of the study (at the end of the 16th week), Ferriman–Gallwey scoring, ovarian volume, laboratory tests, anthropometric measurements, and blood pressure were re-evaluated in participants in both groups.

### Statistical analysis

The study data was analyzed using the statistical package for social sciences (SPSS) software version 21 (SPSS Inc, Chicago, IL). The normality of the quantitative variables was determined using the Kolmogorov-Smirnov test. Normal quantitative variables were described using mean and standard deviation, and non-normal quantitative variables were described using median and interquartile range. Frequency and percentage were also used to describe qualitative data. The Fisher exact test was used to compare qualitative variables between groups and the Mann-Whitney test was used for the comparison of quantitative variables between groups.

Unfortunately, the questionnaires of patients who refused cooperation, or incomplete supplement consumption were mistakenly thrown away. As a result, the data for those patients were lost. This made it impossible to conduct an intention-to-treat (ITT) analysis which includes all randomized participants regardless of protocol deviations or incomplete follow-up. Therefore, a per-protocol analysis was performed. To assess the treatment’s impact on the outcomes, linear regression models were employed, incorporating the effects of treatment, time, and their interaction. This method facilitated a more precise analysis of the treatment’s true effects on the response variable by accounting for potential confounding variables. The assumptions of regression analysis, including the normality of the distribution of the response variable, homogeneity of variance, and independence of observations, were checked respectively by checking the normality of the distribution of the residuals, the graph of the residuals against the fitted values, and the graph of the residuals against time. The level of significance was considered 0.05.

### Ethical considerations

The study was approved by the Ethics Committee of the Gonabad University of Medical Sciences (Code: IR.GMU.REC.1401.080). All participants and their parents or guardians were briefed about the study’s aims and procedures. Then, the willing participants and their parents or guardians gave written informed consent before entering the study.

## Results

**Fig. 1:**
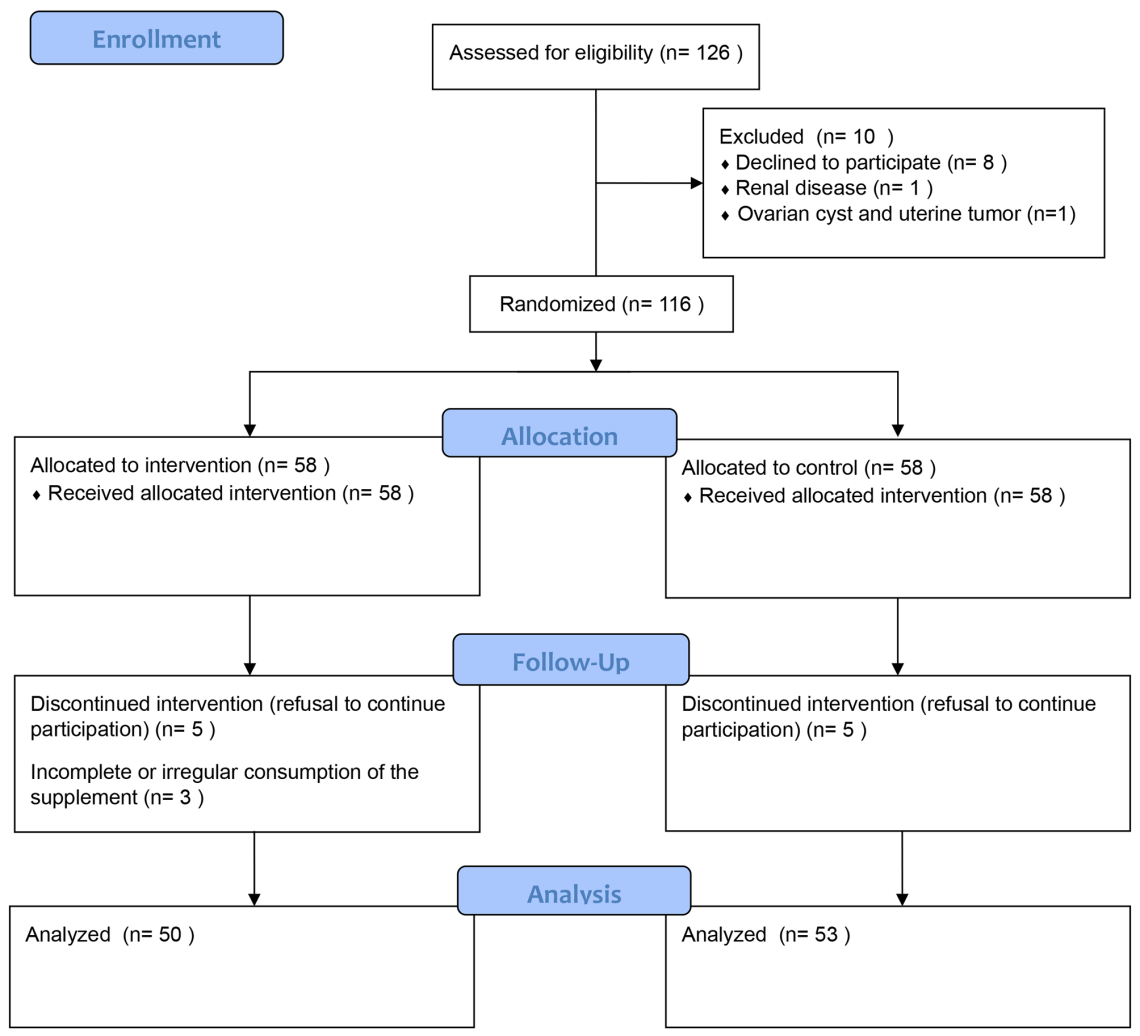
The CONSORT flow diagram of the study

In the current study, of the primary identified 126 adolescent girls with PCOS, 116 entered the study and were randomly assigned to the intervention (*n* = 58) and control (*n* = 58) groups. During the study, five participants from the control group and 8 participants from the intervention group were excluded; therefore, data on 50 participants in the intervention group and 53 participants in the control group were analyzed (Fig. 1).

The demographic and menstrual characteristics of the participants are presented in Table 1. Overall, there were no significant differences between the groups in terms of demographic characteristics, except for paternal education level (*p* = 0.013). These findings indicated a higher percentage of high school diplomas in the control group compared to the intervention group. There were no significant differences between the groups in terms of menstrual characteristics.

**Table 1:** Comparison of the demographic and menstrual characteristics between Nigella sativa and Medroxyprogesterone groups

Variable	Nigella sativa (*n* = 50)	Medroxyprogesterone (*n* = 53)	*p*
**Age (years), Median (Q1, Q3)**	17.0 (16.0, 18.0)	17.0 (16.0, 18.0)	0.418^†^
**Number of siblings, Median (Q1, Q3)**	3.0 (2.0, 4.0)	3.0 (2.0, 4.0)	0.386^†^
**Education level, n (%)**			0.135^#^
Literacy	0 (0.0)	2 (3.8)	
Primary	5 (10.0)	1 (1.8)	
Secondary	32 (64.0)	32 (60.4)	
High school diploma	13 (26.0)	18 (34.0)	
**Maternal occupation, n (%)**			0.407^#^
Housewife	32 (64.0)	30 (56.6)	
Employee	12 (24.0)	20 (37.7)	
Worker	5 (10.0)	2 (3.8)	
Retired	1 (2.0)	1 (1.9)	
**Maternal education, n (%)**			0.207^#^
Primary	18 (36.0)	24 (45.3)	
Secondary	18 (36.0)	10 (18.9)	
High school diploma	4 (8.0)	6 (11.3)	
Diploma	8 (16.0)	13 (24.5)	
Bachelor	1 (2.0)	0 (0.0)	
Master	1 (2.0)	0 (0.0)	
**Paternal occupation, n (%)**			0.284^#^
Unemployed	2 (4.0)	5 (9.4)	
Employee	28 (56)	32 (60.4)	
Worker	15 (30.0)	12 (22.6)	
Retired	5 (10.0)	2 (3.8)	
Freelance	0(0.0)	2 (3.8)	
**Paternal education, n (%)**			**0.013** ^**#**^
Primary	11 (22.0)	9 (17.0)	
Secondary	9 (18.0)	9 (17.0)	
High school diploma	9 (18.0)	22 (41.5)	
Diploma	17 (34.0)	6 (11.3)	
Bachelor	2 (6.0)	7 (13.2)	
Master	1 (2.0)	0 (0.0)	
**Birth order, n (%)**			0.437^#^
1	4 (8.0)	1 (1.9)	
2	14 (28.0)	14 (26.4)	
3	20 (40.0)	20 (37.7)	
4	9 (18.0)	16 (30.2)	
5	3 (6.0)	2 (3.8)	
**Age at menarche (years), Median (Q1, Q3)**	13.0 (12.0, 14.0)	13.0 (12.0, 14.0)	0.555^†^
**Menstrual cycle duration (days), Median (Q1, Q3)**	40.0 (40.0, 45.0)	40.0 (40.0, 45.0)	0.742^†^
**Menstrual bleeding duration (days), Median (Q1, Q3)**	3.0 (2.0, 4.0)	2.0 (2.0, 3.0 )	0.136^†^
**Menstrual bleeding severity, Median (Q1, Q3)**	1.0 (1.0, 1.0)	1.0 (1.0, 1.0)	0.349^†^
**PCOS diagnosis duration, Median (Q1, Q3)**	6.0 (4.0, 7.0)	5.0 (4.0, 7.0)	0.184^†^
**History of dysmenorrhea**			0.233^##^
Yes	2 (4.0)	0 (0.0)	
No	48 (96.0)	53 (100.0)	

Q1, 1st quartile; Q3, 3rd quartile; ^†^ The Mann-Whitney test; ^#^ the chi-square test not exact test; ^##^ The Fisher exact test

A comparison of imaging and laboratory measurements between groups is presented in Table 2. A significant treatment effect was observed for endometrial thickness (*p* < 0.001), LH (*p* = 0.013), BMI (*p* < 0.001), and systolic pressure (*p* = 0.035), indicating a significant difference between the groups in these outcomes before interventions.

**Table 2:** Comparison of imaging and laboratory measurements between Nigella sativa and Medroxyprogesterone groups

Variable		Nigella sativa Mean (SD)	Medroxyprogesterone Mean (SD)	Treatment	Time	Time* Treatment
**Right ovarian volume (cc)**	**Before**	11.96 (2.94)	11.95 (2.34)	< 0.770	0.213	0.002*
	**After**	9.40 (2.12)	11.17 (2.09)			
**Left ovarian volume (cc)**	**Before**	11.28 (3.67)	11.91 (3.07)	0.316	0.343	0.010*
	**After**	8.36 (2.68)	10.84 (3.29)			
**Endometrial thickness (cm)**	**Before**	10.24 (3.15)	9.01 (1.48)	< 0.001*	0.317	0.002*
	**After**	7.30 (2.04)	7.78 (1.55)			
**DHEA (ng/dL)**	**Before**	123.74 (36.49)	125.02 (29.07)	0.736	0.232	0.074
	**After**	113.91 (28.11)	126.44 (28.88)			
**DHEA-S (ng/dL)**	**Before**	128.79 (39.65)	133.98 (35.44)	0.628	0.559	0.120
	**After**	113.89 (31.10)	131.96 (34.28)			
**Testosterone (ng/dL)**	**Before**	0.56 (0.49)	0.66 (0.67)	0.369	0.040*	0.001*
	**After**	0.33 (0.27)	0.65 (0.69)			
**Hirsutism severity score**	**Before**	3.98 (1.56)	3.75 (1.40)	0.111	0.001*	< 0.001*
	**After**	1.36 (0.85)	3.38 (1.79)			
**LH (mU/dL)**	**Before**	15.72 (3.19)	14.29 (3.40)	0.013*	0.004*	< 0.001*
	**After**	9.90 (2.06)	13.57 (2.87)			
**BMI (kg/m** ^**2**^ **)**	**Before**	25.56 (3.63)	23.84 (2.61)	< 0.001*	0.517	0.252
	**After**	24.57 (2.36)	23.72 (2.47)			
**WHR**	**Before**	0.92 (0.06)	0.89 (0.06)	0.064	0.768	0.814
	**After**	0.92 (0.07)	0.90 (0.07)			
**SBP (mmHg)**	**Before**	105.10 (5.67)	103.30 (5.37)	0.035*	0.700	0.610
	**After**	105.04 (4.69)	103.28 (4.24)			
**DBP (mmHg)**	**Before**	71.10 (2.73)	71.51 (2.87)	0.548	0.433	0.330
	**After**	71.66 (2.27)	71.55 (2.51)			

DHEA: dehydroepiandrosterone, DHEA-S: dehydroepiandrosterone sulfate, LH: Luteinizing Hormone, BMI: Body Mass Index, WHR: Waist-hip ratio, SBP: Systolic Blood Pressure, DBP: Diastolic Blood Pressure

The repeated measures analysis of variance was used for the analysis

* Significant effect

Furthermore, a significant effect was noted for the interaction of time and treatment regarding right ovarian volume (*p* = 0.002), left ovarian volume (*p* = 0.010), endometrial thickness (*p* = 0.002), Testosterone (*p* = 0.001), hirsutism severity score (*p* < 0.001), and LH (*p* < 0.001). This suggests a significant difference between the groups in terms of these outcomes from before to after the interventions, after adjusting for differences before the intervention.

Overall, a significantly higher reduction in mean ovarian volume on both sides, endometrial thickness, serum testosterone, hirsutism score, and serum LH were identified in the intervention group compared to the control group.

**Fig. 2:**
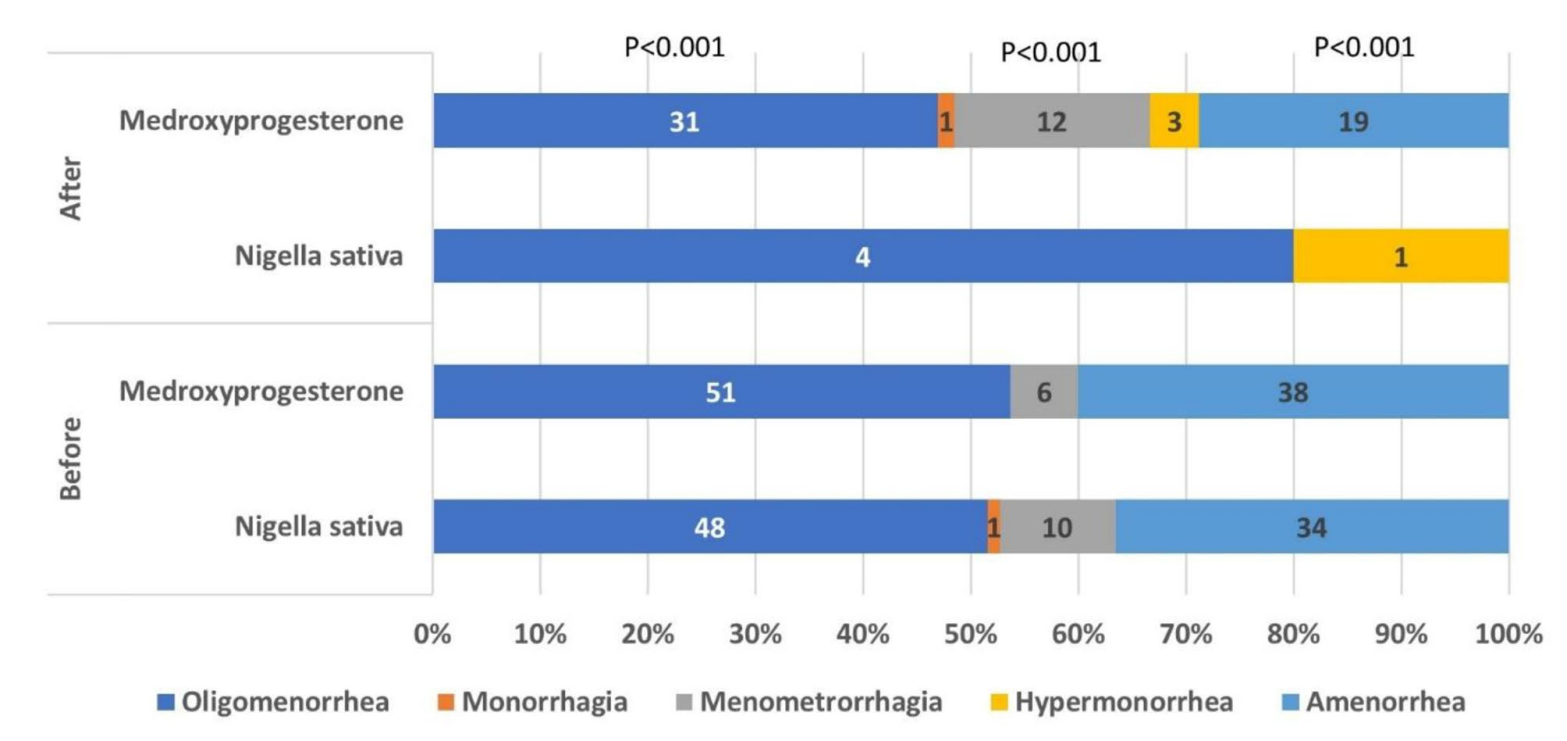
Frequency of menstrual disorders in study groups before and after the study (The percentage of each menstrual disorder in each study group is presented in the horizontal axis. The frequency of each menstrual disorder in the study group is presented on the bar with the same color. There was a significant difference in the frequency of oligomenorrhea, menometrorrhagia, and amenorrhea between groups after the study (*p* < 0.001 for each disorder))

Comparison of the frequency of menstrual disorders between the study groups before and after the study is presented in Fig. 2. (Fig. 2). There was no significant difference between groups in terms of the frequency of menstrual disorders before the study (*p*s > 0.05). There was a significant difference in the frequency of oligomenorrhea, menometrorrhagia, and amenorrhea (*p*s < 0.001) between groups after the study. This finding indicated that the frequency of these menstrual disorders was significantly lower in the intervention group compared to the control group.

## Discussion

The current study was conducted to compare the effects of 16 weeks of Nigella sativa supplementation on ovarian volume, hormonal, and menstrual disorders with those of medroxyprogesterone administration in adolescent girls with PCOS. The results of the current study showed that Nigella sativa supplementation resulted in a significantly higher reduction in ovarian volume, endometrial thickness, serum testosterone, and LH; as well as some menstrual disorders compared to the medroxyprogesterone group.

Similar to the results of the current study in terms of ovarian volume reduction, an animal study showed that intraperitoneal administration of Nigella sativa for three weeks decreased the number of primordial follicles, primary and secondary follicles, and ovarian weight compared to the control group [25].

The results of the current study in terms of the effect of Nigella sativa supplementation on serum testosterone were in line with the findings of previous studies. In a study on rats with PCOS, the combination of Nigella sativa and honey significantly reduced serum testosterone [20]. Another animal study on letrozole-induced PCOS in mice model showed that Nigella sativa administration for 8 weeks significantly reduced serum testosterone levels [26].

To the best of our knowledge, no study has yet investigated the effect of Nigella sativa supplementation on hirsutism. However, similar to the present study, some studies have shown that progesterone administration reduces hirsutism [27, 28]. Women with PCOS often suffer from skin disorders, including hirsutism due to excessive environmental androgens [15]. It seems that the mechanism of Nigella sativa effect on hirsutism might be related to the male sex hormone-reducing effects of Nigella sativa.

The results of the current study in terms of the effects of Nigella sativa supplementation on serum LH levels were similar to the findings of previous studies. A study conducted on rats with PCOS, that showed a high dose of Nigella sativa and honey administration significantly reduced serum LH [20]. The mechanism for this effect can be attributed to the effects of Nigella sativa on insulin resistance. Previous evidence has shown that increased insulin levels and insulin resistance intensify the production of sex steroids stimulated by ovarian gonadotropins and cause abnormal LH secretion [20].

The results of the current study in terms of the effects of Nigella sativa supplementation on oligomenorrhea, menometrorrhagia, and amenorrhea were in line with the findings of some previous studies. For instance, a previous study showed that 16-week Nigella sativa supplementation significantly reduced menstrual cycle intervals and increased menstruation frequency in women with PCOS [29]. Another study showed that oral Nigella sativa supplementation (1000 mg) reduced the severity of physical symptoms of premenstrual syndrome [30]. The exact mechanism of this effect is not known; however, it seems that these effects are due to the phytoestrogen compounds in Nigella sativa extract [31]. Phytoestrogens are weak estrogenic compounds that bind to estrogen receptors and present their agonistic and antagonistic effects, both of which can affect menstrual disorders in PCOS [29]. Another mechanism of the effect of Nigella sativa might be related to its unsaturated fatty acid content, including linoleic acid and oleic acid. The estrogenic effects of these unsaturated fatty acids have been reported in previous studies [32]. This mechanism was hypothesized based on the findings of previous animal studies. A previous study on postmenopausal rats reported that the methanolic extract of Nigella sativa had similar effects to conjugated estrogen on vaginal epithelial recovery [33]. Other animal studies showed that Nigella sativa administration increased the weight of the uterus and serum level of estradiol [33, 34].

Other mechanisms for the potential effect of Nigella sativa on the improvement of oligomenorrhea include the anti-inflammatory and antioxidant properties of this plant compound [35]. Recent studies have emphasized the important role of oxidative stress in the pathogenesis of PCOS and suggested the effectiveness of antioxidant consumption in improving these symptoms [36]. The antioxidant mediators of Nigella sativa have been studied using enzymatic methods in previous studies [37]. On the other hand, oxidative stress can result in the production of inflammatory mediators, including interleukin-6 and tumor necrosis factor-alpha, and affect the ovary and endothelium causing anovulation and premature atherosclerosis in PCOS [38]. Inflammatory mediators are produced in the initial responses of the immune system to obesity and insulin resistance in patients with PCOS, which may be reduced by antioxidants [39].

The results of previous studies in terms of the effects of Nigella sativa supplementation on BMI and WHR were contradictory. For instance, a previous study showed that Nigella sativa supplementation along with exercise decreased BMI, waist circumference, and WHR in young overweight women [40]. A systematic review showed that Nigella sativa supplementation reduced BMI but not WHR [41]. These contradictory results seem to be due to the difference in the study population (overweight women compared to PCOS adolescents in our study) and the difference in the intervention (Nigella sativa with exercise compared to Nigella sativa alone in our study).

The results of the previous studies in terms of the effects of Nigella sativa supplementation on systolic and diastolic blood pressure were contradictory. A previous study showed that hydroalcoholic extract of Nigella sativa for 8 weeks significantly decreased systolic and diastolic blood pressure in a dose-dependent manner among patients with mild hypertension [42]. Furthermore, in another study on hypertensive patients, Nigella sativa supplementation for 12 weeks significantly reduced blood pressure [43]. The reason for the contradiction in the findings can be due to the inclusion of hypertensive patients in the mentioned study compared to normotensive adolescents in the current study.

The current study showed that although the mean DHEA and DHEA-S decreased after the intervention, but the difference was not statistically significant. In this regard, the findings of the previous studies were contradictory. For instance, an animal study showed that the administration of Nigella sativa combined with honey to male rats increased DHEA and androstenedione [44]. In addition to the fact that this study is animal-based, the contradiction in the findings can be caused by the consumption of the combination of honey and Nigella sativa. It is also possible that increasing the dosage of black seeds can cause different therapeutic effects.

The study faced a limitation of missing data for patients who were lost to follow-up due to discarded questionnaires when they discontinued participation or had irregular supplement consumption. This resulted in the inability to perform an intention-to-treat (ITT) analysis, which could have provided a more comprehensive assessment of the intervention’s effectiveness. So, the impact of missing data on the study results should be considered when interpreting the findings. Further studies are needed to confirm the findings of our study.

## Conclusion

The results indicated that Nigella sativa supplementing could reduce ovarian volume, improve hormonal balance, and alleviate menstrual irregularities in adolescents with PCOS compared to medroxyprogesterone administration. However, these findings should be confirmed by further studies.

### Electronic supplementary material

Below is the link to the electronic supplementary material.

Supplementary Material 1

Supplementary Material 2

## Data Availability

The datasets generated and analysed during the current study are not publicly available but are available from the corresponding author on reasonable request.

## Abbreviations

PCOS: Polycystic ovary syndrome
DHEA: Dehydroepiandrosterone
DHEA-S: Dehydroepiandrosterone sulfate
LH: Luteinizing hormone
BMI: Body mass index
WHR: Waist-hip ratio
SPSS: Statistical package for social sciences
SBP: Systolic Blood Pressure
DBP: Diastolic Blood Pressure
