# Supplementary material for: The possible short-term of Nigella sativa – L in the management of adolescent polycystic ovarian syndrome: results of a randomized controlled trial
Source: J Ovarian Res. 2024 Jul 12;17:144. doi: 10.1186/s13048-024-01460-x (PMC11242022; doi:10.1186/s13048-024-01460-x)

**Product shape**

1000 mg soft capsule

Standardized based on the presence of at least 6.5 mg of thymoquinone and 495 to 605 mg of linoleic acid in each soft capsule.

**Product components**

black seed oil

**Active ingredient**: Thymoquinone, Dithymoquinone, Thymohydroquinone, Nigellamin, Nigellone, P-cymen, unsaturated fatty acids such as linoleic acid and oleic acid.

**Possible side effects**

No specific side effects have been observed.

**Method of Use**

Take one soft capsule daily with some liquid.


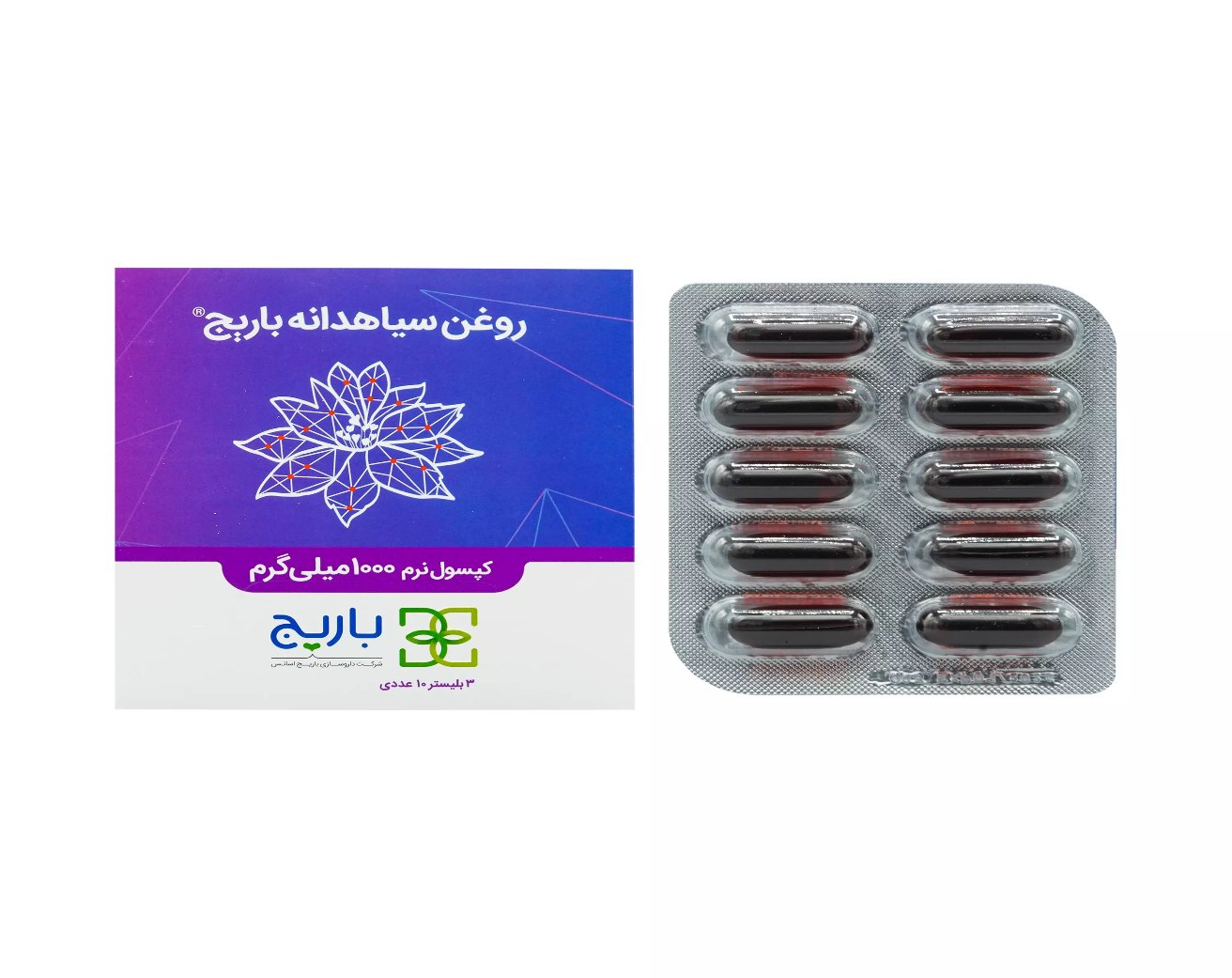

Supplement: Supplementary file 2 — Supplementary Material 2 [file 13048_2024_1460_MOESM2_ESM.docx]
